# hERG-toxicity prediction using traditional machine learning and advanced deep learning techniques

**DOI:** 10.1016/j.crtox.2023.100121

**Published:** 2023-09-01

**Authors:** Erik Ylipää, Swapnil Chavan, Maria Bånkestad, Johan Broberg, Björn Glinghammar, Ulf Norinder, Ian Cotgreave

**Affiliations:** a Computer Systems Unit, Research Institutes of Sweden RISE, Kista 164 40, Sweden; b Unit of Chemical and Pharmaceutical Toxicology, Research Institutes of Sweden RISE, Södertalje 151 36, Sweden; c Department of Computer and Systems Sciences, Stockholm University, Kista 164 07, Sweden; d Preclinical Development & Translational Medicine, Swedish Orphan Biovitrum AB, Solna 171 65, Sweden

**Keywords:** Deep Learning, Graph-neural Network, hERG Channel, Random Forest, Recurrent-neural Network, Support-vector Machines

## Abstract

The rise of artificial intelligence (AI) based algorithms has gained a lot of interest in the pharmaceutical development field. Our study demonstrates utilization of traditional machine learning techniques such as random forest (RF), support-vector machine (SVM), extreme gradient boosting (XGBoost), deep neural network (DNN) as well as advanced deep learning techniques like gated recurrent unit-based DNN (GRU-DNN) and graph neural network (GNN), towards predicting human ether-á-go-go related gene (hERG) derived toxicity. Using the largest hERG dataset derived to date, we have utilized 203,853 and 87,366 compounds for training and testing the models, respectively. The results show that GNN, SVM, XGBoost, DNN, RF, and GRU-DNN all performed well, with validation set AUC ROC scores equals 0.96, 0.95, 0.95, 0.94, 0.94 and 0.94, respectively. The GNN was found to be the top performing model based on predictive power and generalizability. The GNN technique is free of any feature engineering steps while having a minimal human intervention. The GNN approach may serve as a basis for comprehensive automation in predictive toxicology. We believe that the models presented here may serve as a promising tool, both for academic institutes as well as pharmaceutical industries, in predicting hERG-liability in new molecular structures.

## Highlights

• Robust AI/ML models for the largest hERG dataset to date.
• Benchmarking of advanced deep learning techniques against traditional techniques.
• GNN was found to be a winning model.
• GNN is free of any feature engineering steps and has minimal human interventions.
• GNN may serve as a basis for comprehensive automation of predictive toxicology.

## Graphical abstract

**Figure:**
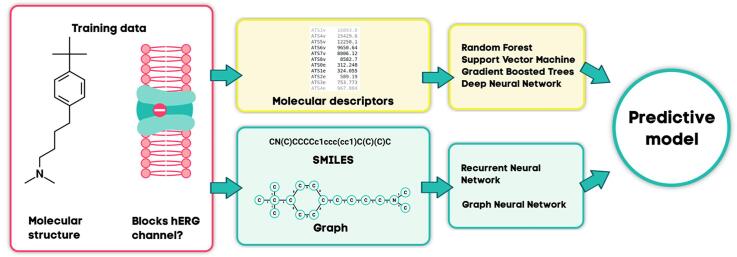

## Introduction

The human heart consists of specialized cells that have their own capability to generate automatic action potential for the heart to contract rhythmically. There is a complex, organized movement of charged ions across myocardial cell membranes through a wide variety of specific ion channels, ultimately resulting in the generation of the cardiac action potential (de Carvalho et al., 1969). Cardiac action potentials consist of five phases: Rapid depolarization (phase 0), early repolarization (phase 1), the plateau phase (phase 2), repolarization (phase 3), and the resting phase (phase 4) (Grant, 2009). In phase 0, there is rapid sodium ion influx through sodium channels which drives rapid depolarization (contraction). For phase 1, transient potassium channels open, leading to efflux of potassium ions, resulting in early repolarization (relaxation). In phase 2, there is outward movement of potassium via delayed rectified K + channels which are encoded by a gene called human ether-á-go-go-related gene (hERG) (Sanguinetti et al., 1995). Blockade of this voltage dependent K + ion channel (hERG, KCNH2, Kv11.1) by drugs and other chemicals lead to delayed depolarization and so-called Q-T interval prolongation. In its most severe form, this can lead to a condition termed torsade de pointers (TDP), which is life threatening and many drugs have been withdrawn from the market for this reason (Brown, 2004).

As per the International Conference of Harmonization (ICH) guideline (S7B), there is need for testing hERG sensitivity and cardiac safety pre-clinically for every new drug (Shah, 2005). As a consequence, assessment of hERG-related cardiotoxicity liability has become a crucial step in drug early discovery. Several *in vitro* assays have been developed for pre-clinical evaluation of hERG-related cardiotoxicity, e.g., fluorescence-based assays (Dorn et al., 2005), rubidium-flux assays (Chaudhary et al., 2006), radioligand binding assays (Angelo et al., 2003), *in vitro* electrophysiology measurements (Tao et al., 2004), flow cytometric assay (Kanner et al., 2018), and other high throughput assays (Titus et al., 2009). Before testing new candidates in these costly assays, many now conduct pre-testing using *in silico* models to narrow down candidates. Several *in silico* models have to date been developed using different traditional machine learning (ML) approaches such as multiple linear regression (MLR) (Pourbasheer et al., 2013), partial least square (PLS) (Su et al., 2010), random forest (RF) (Wisniowska et al., 2010), support vector machines (SVM) (Yap et al., 2004, Ogura et al., 2019), naive Bayes (Sun, 2006), k-nearest neighbor algorithm (k-NN) (Gunturi et al., 2008, Chavan et al., 2016), and deep neural networks (DNN) (Koutsoukas et al., 2017, Cai et al., 2019, Zhang et al., 2019, Choi et al., 2020, Ryu et al., 2020).

However, all above mentioned models have been solely derived using traditional molecular descriptors or fingerprints for encoding structures. The performance of any ML model is heavily reliant on the selection of input features (data representation). Such features are usually pre-selected by applying prior human knowledge or statistical operations. This type of feature engineering has effectively supported ML algorithms in building robust models. However, these methods rely on devising the right predictive features for a task, since the learning algorithm will only be able to use the information in the descriptors when solving a problem. If the right features are not available, the algorithms cannot learn to solve the problem better than by “guessing”. On the contrary, to make progress towards artificial intelligence (AI), it is highly desirable to make ML algorithms less dependent on feature engineering, with a minimum of human intervention (Bengio et al., 2013). An AI model can only be effective if it can identify and extract meaningful information and hidden factors in the given input data. Advanced deep learning is a new paradigm in ML where useful features are learnt from the complete representation we have of the observations. Instead of using manually crafted functions that filter the inputs to some descriptors, which are hopefully predictive of the task, advanced deep learning techniques can automatically learn to extract precisely the right features using observations alone. This can be thought of as a descriptor-free approach that has gained popularity in recent years.

In the case of hERG liability predictions, some attempts have been made to employ advanced deep learning techniques through graph convolutional neural network (GCNN) (Cai et al., 2019, Hu et al., 2019, Ryu et al., 2020, Wang et al., 2020), self-attention based DNN (Kim and Nam, 2020), recurrent neural networks (RNN) (Siramshetty et al., 2020), and GGNN-coupled RNN (Yang et al., 2020). Among all of these AI-based hERG models, only the study by Hu *et al*. (Hu et al., 2019*)* has, to our knowledge, used the currently largest and publicly available training dataset. However, the authors did not benchmark their approach against other traditional ML and advanced deep learning approaches. Therefore, our study aimed to employ a series of traditional ML and advanced deep learning techniques to build hERG toxicity prediction models using this large hERG dataset (Ogura et al., 2019), and benchmark these techniques.

## Materials and methods

### Data set

The Ogura *et al.* study in 2019 compiled four major hERG datasets to form a largest dataset, comprising of in total 291,219 compounds (Ogura, 2019, Ogura et al., 2019). This dataset is based on integrated hERG dataset by Sato *et al.* study (Sato et al., 2018) where authors have curated hERG-related *in vitro* records from hERG central, PubChem, ChEMBL, and GOSTAR databases. Sato *el al.* further formatted activity types (IC_50_, EC_50_, ED_50_, K_i_, K_d_) of the collected records to an IC_50_ type. Lastly, the compounds were discretized into hERG positive class if showed IC_50_ less than equal to 10 uM or percentage inhibition greater than equal to 50%. Ogura *et al.* study had divided this dataset into training (203841) and test set (87361) to train and validate their model. For our study, we have used same data from the Ogura *et al.* study (Ogura, 2019). Retrieved from the supplementary file of Ogura *et al.*’s study, a total of 203,853 compounds were employed for model training and validation, whereas the test set of 87,366 compounds was used to test the predictivity of the resulting models. We have used a third party cheminformatics toolkit called RDKit (Landrum, 2013), to parse the SMILES. This library was not able to parse some SMILES, which were then discarded from the respective sets.

### Descriptors

A total of 1613 numerical, 2-dimensional (2D) features were derived using the Mordred python library (Moriwaki et al., 2018). For the purpose of ensuring consistent data for the model building, we have refrained from imputing any missing information to our feature set. Therefore, some 427 features with missing or infinite values were discarded. The remaining 1186 features were used for construction of models described below in Section "Model construction". The mean values of the 1186 selected features were calculated to fill missing values during testing on new compounds.

### Input for advanced deep learning techniques

Neural networks were operated by sequentially transforming inputs (in the form of numeric vectors) to a desired output (here, toxic/non-toxic category). For fixed dimensional continuous inputs this poses no challenge, but for inputs which are categorical we need to represent these variables using continuous values, typically vectors. In the ML literature, this vector representation of a categorical value is referred to as an “embedding”.

The advanced deep learning methods utilized in the current study are different kinds of deep neural networks. The representation of molecules should be unfiltered, that is they should be in a form which preserves all information available in the original data. For the Gated Recurrent Unit coupled deep neural networks (GRU-DNN), this representation is in the form of SMILES notation, while for the graph neural networks (GNN) it is represented as a mathematical graph.

#### Structural representation as SMILES

**Fig. 1:**
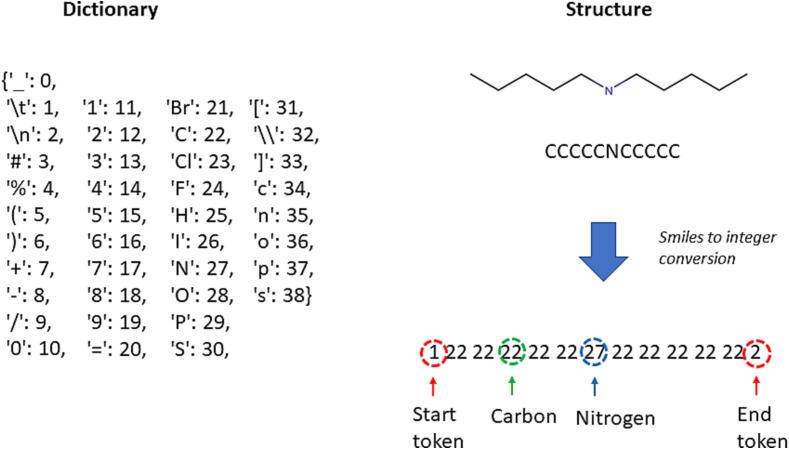
Illustration of SMILES to integer vector conversion for one of the training set compound.

From a ML perspective, a SMILES is considered a sequence of tokens. For RNN, it is customary to add extra symbols as control symbols to the network, so it can learn where a molecule starts and where a molecule ends, since this is not part of the SMILES representation. In this work, we tokenized the SMILES on a per-character level, i.e. each character becomes its own token. A dictionary of all characters occurring in the dataset was created, where each token was associated with a unique integer code (see Fig. 1). A simple coding scheme which assigns a unique integer to permissible character in a SMILES was used. These codes were then used to lookup the corresponding embedding vectors for each symbol which is the input to the neural network. This process is applied to all tokens of a SMILES sequence, resulting in a sequence of embedding vectors.

#### Structural representation as graph

**Fig. 2:**
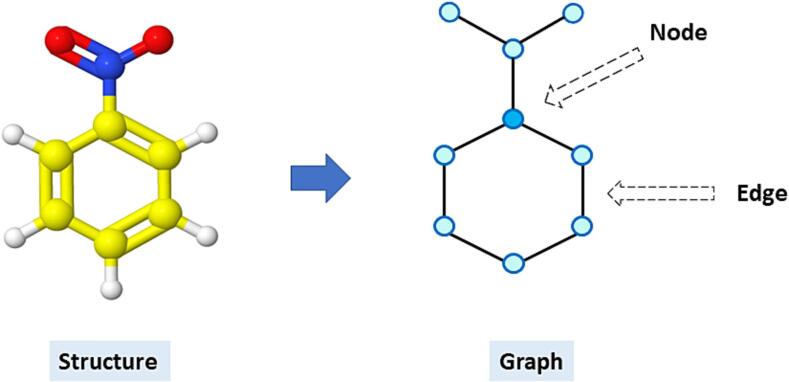
Depiction of a graph, where an atom is represented as a “Node”, and a bond is represented as an “Edge”.

In the case of GNN, molecules were represented as a mathematical graph, an object that can represent binary relationships between arbitrary objects, which is used heavily in network theory, and as a representation of molecules. Here the atoms correspond to nodes in the graph, and bonds correspond to edges (see Fig. 2). The atomic features such as atom type, implicit and explicit valence, whether the atom is part of an aromatic ring, the formal charge and degree are represented as embeddings (referred to as node embeddings). Similarly, bond features such as bond types, if it is conjugated and whether it is part of a ring are also represented as embeddings, which we refer to as edge embeddings. As input to the GNN each molecule was then represented by its set of node embeddings and edge embeddings for each pair of bonded atoms in the graph. These embeddings are dense vectors of free parameters which are part of the GNN models and optimized with them.

### Model construction

#### Subsampling

**Fig. 3:**
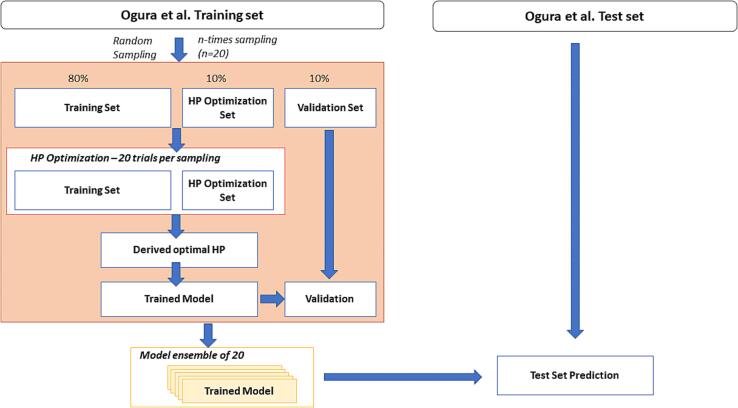
Subsampling scheme used for training the 20 models.

For all learning algorithms, the same sampling schemes were used to construct a set of 20 separate models. A total of 203,853 training set compounds were used for the model building and validation. We employed a subsampling technique, where the models were constructed using randomly sampled 80% compounds from the training set. Of the remaining data, 10% was used as a validation set for the hyper parameter optimization and the left over 10% was used to estimate generalization performance of the final model. This procedure was then repeated 20 times per learning algorithm to produce a diverse set of models (refer Fig. 3). In order to make our procedure fully reproducible, we have set a unique seed at each subsampling event using three different python libraries i.e. torch, numpy and random. The final 20 models were then externally validated using the test set of 87,366 compounds.

#### Hyper parameter optimization

For each learning algorithm and each subsampled dataset, an independent hyperparameter optimization study was performed. Hyperparameter search was performed using Optuna python library, employing Tree-structured Parzen Estimators as the optimization model (Akiba et al., 2019). The Tree-Structured Parzen Estimator is a Bayesian optimization technique which sorts hyperparameter values into two groups based on a predefined quantile value. These two groups are then modeled using the Parzen Estimator, where the hyperparameters are located based on highest expected improvement. The hyperparameters with the highest expected improvement are further evaluated using same procedure until the given number of trials. In our case, each hyper parameter optimization study was performed for 20 trials. The number of hyper parameters that were optimized ranged from two to seven parameters, depending on the algorithm. For each algorithm the set of hyper parameters which performed best in terms of AUC ROC score were subsequently used to train the final model on the 80% subsampled data plus the 10% data that was used as validation dataset during hyper parameter optimization.

In summary, a total of 420 models were trained per algorithm, of which 400 were used for determining optimal hyperparameters. The remaining 20 models were then trained using optimal hyperparameters identified in the previous step, one for each subsampled training dataset.

#### Discretization threshold

To calculate several classification metrics based on the confusion matrix, the model’s prediction score needs to be discretized to a binary class. Therefore, we needed to decide on which threshold to use for such discretization. Naively using 0.5 as the threshold can leads to an incorrect prediction, since most of the models do not learn to calibrate their output probabilities (despite an imbalanced dataset). To make the comparison fair between models, the discretization threshold was optimized per model using the 10% validation set of that subsample and by applying Youden’s J-statistic (Schisterman et al., 2008). Choosing the right threshold shall give the optimal balanced accuracy for the given validation set. Note that this is not an issue for the ROC AUC score, which essentially is designed to be agnostic to any specific threshold and is the metric used to compare models in this paper.

#### Random forest (RF)

A random forest classifier (RFC) is a supervised, non-parametric ML algorithm that is used for classification (Ho, 1995). RFC creates a random forest, which is an aggregation of different decision trees. To train the model, hyper parameter search was performed over number of estimators, splitting criterion, max depth and max number of leaf nodes. Minimum samples to split an internal node equal to 2, minimum sample to be at external node equal to 1, and the number of variables that were to be considered at each split (mtry) equals the square root of total number of features i.e. 34. Since the training data was imbalanced, we employed the ‘balanced’ class weight feature from SCIKIT-LEARN library (Pedregosa et al., 2011).

#### eXtreme gradient boosting (XGBoost)

The gradient boosted tree is a supervised ML method, with built-in decision trees for regression and classification (Hastie et al., 2009). While the RFC takes the average of many decision trees with bagging, the gradient boosted classifier instead combines the trees with gradient boosting. In boosting, the trees are built sequentially such that each subsequent tree aims to minimize the errors of the previous tree. Each tree learns from its predecessors and updates the residual errors. We use the boosting system XGBoost, which is designed to be highly efficient and widely used in applied ML (Chen and Guestrin, 2016).

For the XGBoost model, early stopping was employed using a patience of 10 rounds and a maximum of 500 boosting iterations. Hyperparameter optimization was performed on the learning rate and maximum tree depth of the ensemble. The training was performed using GPU, which had limited the tree method to ‘gpu_hist’.

#### Support vector Machine (SVM)

A support vector machine (SVM) is a supervised ML technique which was proposed by Vapnik et al. (Boser et al., 1992). SVM is one of the most widely used technique for classification purpose. An SVM can be applied to both linearly separable and non-linearly separable data. In case of non-linear data, SVM employs a kernel function that transforms data to a new higher dimensional space where the data can be linearly separated. The polynomial, radial basis function (RBF), and sigmoid function are common kernels used for SVM. We used the RBF kernel since it has fewer parameters and has been found effective in modeling several endpoints. This kernel has two parameters which need to be determined i.e., cost and gamma. The cost trades off misclassification of training compounds to minimize the model's error, while parameter gamma defines the non-linear mapping from input space to the new higher dimensional feature space. Both these hyperparameters were optimized like the other algorithms using Optuna library. The classifier was trained using the thundersvm library with “balanced” class weights. The classifier was also trained to output a prediction score using platt scaling.

#### Deep learning common training protocol

The training protocol was shared between the three deep learning algorithms: DNN, GRU-DNN and GNN. They were all trained by minimizing binary cross entropy using mini-batch stochastic gradient descent, with mini-batches of size 512. The ‘AdamW’ optimization algorithm was used, which was configured with an optimal learning rate (LR) per hyper optimization study (obtained from hyper parameter optimization as described in Section "Hyper parameter optimization"), a weight_decay rate of 10^-6^, with default values for beta1 of 0.9, beta2 of 0.999 and epsilon of 10^-8^. As our data was imbalanced, we implemented ‘WeightedRandomSampler’ feature from the PyTorch library (Paszke et al., 2019). This feature randomly over samples the minority class for each mini-batch to balance the number of examples from each toxicity class that the model observes in each batch.

The models were trained for at most 100 epoch, where an epoch is one full iteration run throughout all training data. We employed early stopping criteria of maximum validation of the AUC ROC score, with a patience value of 20 epochs. Thus, if there was no improvement seen in the monitored validation AUC ROC scores within 20 epochs, model training was stopped, and the optimal model was selected that had achieved maximum AUC ROC score for the validation set. During training, a learning rate schedule was employed, where the learning rate was decreased by a factor of 0.1 if no improvement was seen on the validation set after 10 epochs.

#### Deep neural networks (DNN)

Deep learning is a subfield of representation learning, where complex input features are automatically transformed to a data representation in which the problem can be more easily solved (Bengio et al., 2013). Feed-forward neural network (FFNN, also known as DNN) is the simplest variant of deep learning. The DNNs trained in this work were fully connected using Rectified Linear Units (ReLU) as nonlinearities. The architecture of the network was independently determined for each of the 20 subsamples using hyper parameter optimization. The parameters optimized were learning rate, dropout rate, number of layers and size of layers.

#### Gated recurrent unit coupled deep neural networks (GRU-DNN)

Recurrent Neural Networks (RNN) is a special type of neural network that is created for sequential data (www.tensorflow.org). RNN possesses an internal memory, by which it can remember vital information from the input it receives (Azzouni and Pujolle, 2017). Such ability enables it to precisely predict what is coming next in the given sequence. This is how an RNN operates: 1) The RNN is first required to convert an input into a machine-readable numeric vector. 2) Then it applies a nonlinear function (such as composition of a logistic sigmoid with an affine transformation) to the input vector to obtain an output called recurrent hidden state. 3) Finally, for the given position of an element in the input vector, the RNN finds the next possible element (in the form of a probability distribution), which is obtained by applying a multi-class, logistic regression function called softmax to the recurrent hidden state at the given position (Chung et al., 2014). There is one drawback associated with standard RNN, i.e. a vanishing gradient, explained elsewhere (Le and Zuidema, 2016). Therefore, we have implemented the newer generation of RNN called gated recurrent unit (GRU) in our study (Cho et al., 2014).

The GRU is a modified version of RNN (Dey and Salem, 2017). The GRU approach was employed to encode SMILES. In the GRU type of network, there are two gates involved: the update gate and the reset gate. These gates remove irrelevant information and decide what information should be passed to the network output and which information to keep from several previous operations. The tokenized SMILES served as input to the GRU. Then we use the embedding module from PyTorch that converts tokenized smiles to a fixed length, 2D embeddings. The GRU network was implemented using the PyTorch GRU module. The final recurrent state of the GRU network was fed to a dense fully connected layer which produced the final logit for the classification.

The architecture of the network was independently determined for each of the 20 subsamples using hyper parameter optimization. The parameters optimized were learning rate, batch normalization, residual connections, dropout rate, number of layers and size of layers (including embedding layer size).

#### Graph neural network (GNN)

A graph neural network (GNN) is a class of DNN models that, instead of learning from non-structural data, learns from graphs (Zhou et al., 2020). The graph structure makes it possible to use the model on problems where the objects have relations between each other, such as atoms in molecules. A graph consists of objects (nodes) and their relationships (edges). We represented all molecules as graphs (Section "Structural representation as graph"). The inputs to the model were features describing the atoms and the bonds. The model generally learns by passing messages between the neighboring nodes and edges with the help of a local neural network. We stacked multiple of these messages to create a deeper model.

We used the continuous kernel-based convolutional operator which uses the edge and node features to create a weighted sum of the neighboring nodes (Gilmer et al., 2017). We used a two-layer feed-forward neural network to derive the weights, which inputs the edge feature between two atoms (nodes) and their respective node features and outputs a weight. In between each message passing layer we added a normalization layer, a ReLU activation function and a residual connection as proposed in the literature (Li et al., 2019). The model was implemented using the PyTorch Geometric library (Fey and Lenssen, 2019).

The input graph has consisted of atom and bonds with categorical features. These categorical features were embedded separately using PyTorch embedding module, and the resulting embedding vectors were summed per node and edge before they were fed to the graph convolution layers. The output of the graph convolution layers was a set of vectors, one per atom in the input molecule. These were passed through a dense nonlinear layer, after which they were aggregated using global mean pooling, averaging the vector node representations for all nodes in the graph. This resulted in a single vector which was fed to two-layer dense neural network to produce the logit for the binary class prediction. Hyper parameter optimization was used to determine optimal learning rate, normalization layers, residual connections, dropout rate, number of graph convolution layers and size of graph convolution layers.

### Timing predictions

To compare training times vs. prediction time we looked at the top performing models in each of the descriptor-free (GRU-DNN, GNN) and descriptor-based (RF, SVM, DNN, XGBoost) categories. This resulted in two sets of 20 models, where each model was timed on its predictions using a random sample of 50 molecules from the external validation set. The timing included the full prediction, from SMILES representation to class prediction. All predictions were run on the same hardware, an AMD Ryzen 7 5800X 8-Core Processor, Ubuntu Linux computer with an NVIDIA RTX 3090 GPU. The deep learning models were timed both using the GPU and CPU, while the XGBoost model was allowed to run using GPU with 16 workers allocated to descriptor calculations.

### Software and modules

**Fig. 4:**
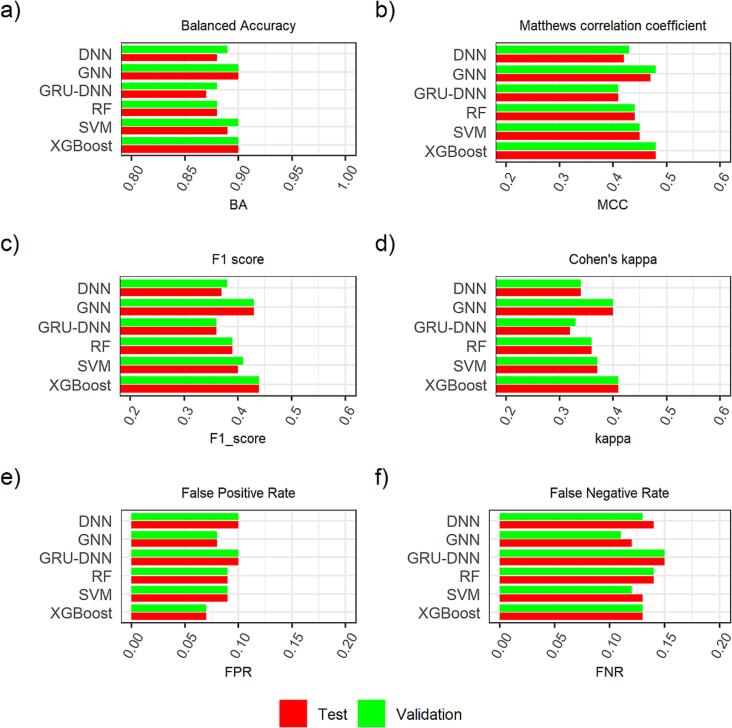
Comparison of all six techniques on the basis of a) BA, b) MCC, c) F1 score, d) Cohen’s kappa, e) FPR and f) FNR, where the test set predictions were performed using winning models from respective techniques.

**Fig. 5:**
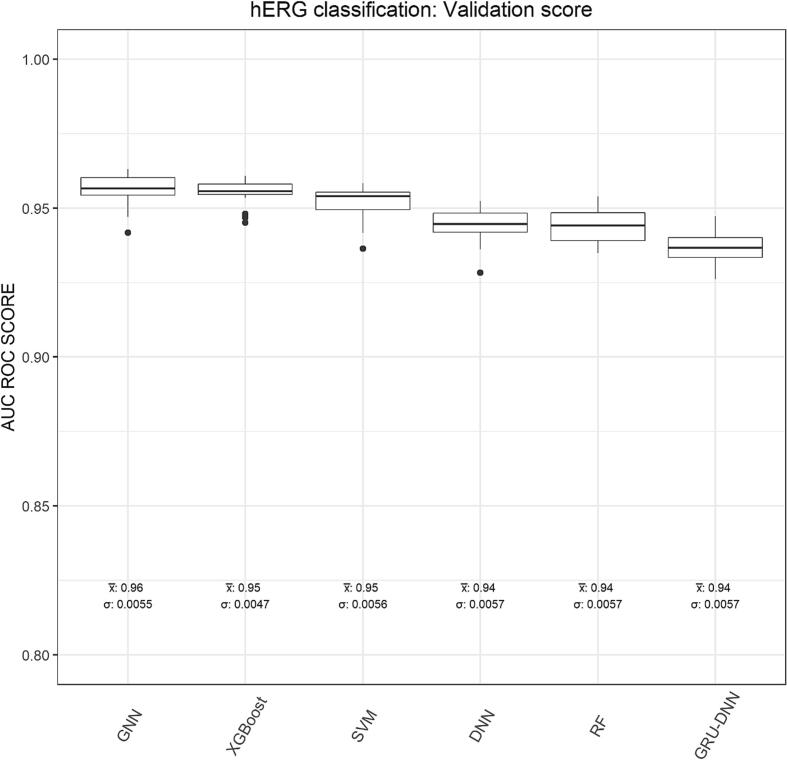
Boxplot explaining mean (x̅) and variation (σ) in the validation set AUC ROC scores for all six techniques.

**Fig. 6:**
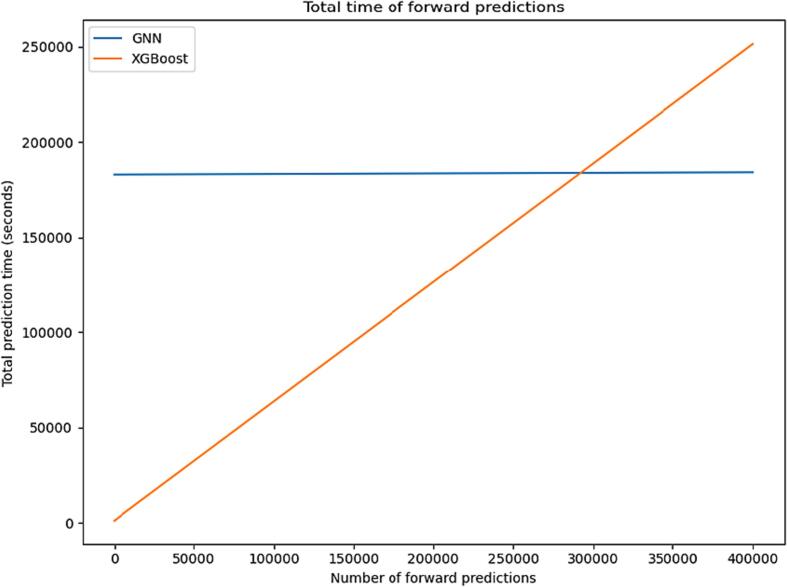
Summary of time comparison for forward prediction with our top performing models.

**Fig. 7:**
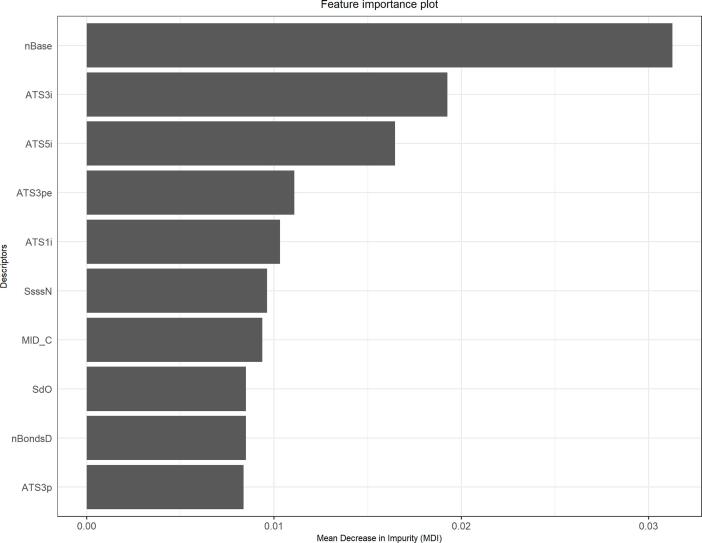
Feature importance plot for 1st RF model.

**Fig. 8:**
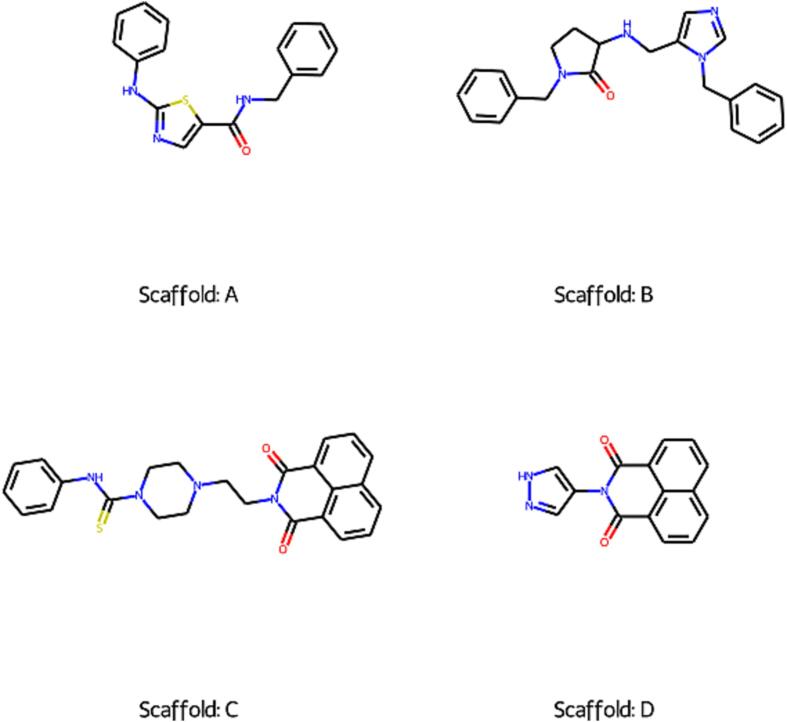
Depiction of four Murcko’s scaffolds observed in some of the test set compounds.

All models were developed using Anaconda version 4.10.1 with Python version 3.9, RDKit version 2022.03.3 and Optuna version 2.10. The descriptor-based models used Mordred version 1.2. The RF model was built with SCIKIT-LEARN library (version 1.1.1) on an Ubuntu Linux platform. The SVM model was built with ThunderSVM library version 0.3.2 using an NVIDIA GeForce GTX 1080ti graphics card on an Ubuntu Linux platform. XGBoost was trained using xgboost version 1.6.2 on an Ubuntu Linux platform using an NVIDIA 3090 GPU. The DNN and GRU-DNN models were built using PyTorch (version 1.11) with a NVIDIA GeForce RTX 3090 on an Ubuntu Linux platform. The GNN models were built with PyTorch and PyTorch Geometric modules (PyTorch version 1.11, pytorch geometric 2.0) with a cuda graphics card (NVIDIA V100) on an NVIDIA DGX Linux platform. Fig. 1, Fig. 2, Fig. 3 were created in MS PowerPoint. Fig. 4, Fig. 5, Fig. 7 were constructed using R (version 3.6.1) in RStudio (version 1.1.456). Fig. 6 was created with Python version 3.9 while Fig. 8 was created with RDKit version 2021.03.4 using python 3.8.8.

## Results and discussion

Predictive performances of all techniques (algorithms) for the validation, as well as test set predictions are listed in Table 1, Table 2, respectively.

**Table 1:** Summary of validation set prediction.

**Technique**	**TN**	**FP**	**FN**	**TP**	**BA**	**AUC ROC score**
GNN	16,960	1444	74	568	0.90	0.96
XGBoost	17,028	1376	81	561	0.90	0.95
SVM	16,803	1601	77	565	0.90	0.95
DNN	16,630	1774	85	557	0.89	0.94
RF	16,792	1612	92	550	0.88	0.94
GRU-DNN	16,530	1874	93	549	0.88	0.94

TN = True Negative, FP = False Positive, FN = False Negative, TP = True Positive, BA = Balanced Accuracy.

**Table 2:** Summary of the test set prediction.

**Technique**	**TN**	**FP**	**FN**	**TP**	**BA**	**AUC ROC score**
GNN	72,868	6125	333	2402	0.90	0.95
XGBoost	73,165	5828	360	2375	0.90	0.95
SVM	72,145	6848	342	2393	0.89	0.95
DNN	71,350	7643	374	2361	0.88	0.94
RF	72,166	6827	396	2339	0.88	0.94
GRU-DNN	70,892	8101	411	2324	0.87	0.93

TN = True Negative, FP = False Positive, FN = False Negative, TP = True Positive, BA = Balanced Accuracy.

### Validation set prediction

The applied subsampling strategy delivered 20 models for each technique, average performance metrics for each technique have been listed in Table 1. For all validation set compounds, each of these models has predicted a probability score for the positive class. These scores were later discretized into predicted classes by using a best threshold approach. Best thresholds for all models for each technique have been listed in **SI** Table 1.

The resulting models for each of the six techniques were evaluated using various statistical measures. The receiver operator characteristic (ROC) curve is a probability plot of sensitivity against false positive rate (i.e. 1-specificity) at different threshold rates. While the area under the curve (AUC) is a summary of ROC curve and is described as the classifier’s ability to distinguish between positive and negative classes. AUC explains the degree of separability i.e. it explains how much the classifier is capable of distinguishing between given two toxicological classes. In other words, the higher the AUC, the better the classifier is at predicting positive compounds as positives and negative compounds as negatives. The AUC ROC score for validation were 0.96 for GNN, 0.95 for both XGBoost and SVM, 0.94 for remaining three techniques i.e. DNN, RF and GRU-DNN.

Among the six techniques, GNN performed best, where it has correctly predicted 568 of 642 positive compounds and 16,960 of 18,404 negative compounds. For the positive class, the GNN technique has performed best, whilst XGBoost technique performed best for the negative class by predicting 17,028 out of the 18,404 negative compounds correctly. Sensitivity describes the classifier’s ability to categorize a positive compound into the positive category, while specificity describes the classifier’s ability to categorize a negative compound into the negative category. For the validation set, the highest sensitivity score was showed by GNN techniques, which was equal to 0.89 (see **SI** Table 2). The validation set specificity score of 0.93 was showed by XGBoost technique, highest among six. Balanced accuracy (BA) is a widely used performance metric, which is defined as an arithmetic mean of sensitivity and specificity. The validation set BA scores for all these six techniques were shown to be in the range of 0.88–0.90 (see Table 1), where the GNN showed the highest and the GRU-DNN showed the lowest score.

The F1-score is the harmonic mean of the precision and recall. The highest F1 score of validation was observed for XGBoost technique, which was equal to 0.44, whilst the lowest F1 score was observed for the GRU-DNN technique which was equal to 0.36 (refer **SI** Table 2). Cohen’s kappa is a chance adjusted index of agreement. In other words, it explains how much better the given classifier is, over the performance of a classifier that guesses at random according to the frequency of each class. The highest validation set kappa was observed for XGBoost technique equal to 0.41 and the lowest for GRU-DNN, equal to 0.33. However, there is a criticism regarding use of F1-score and Cohen’s kappa in that F1-score is an asymmetric metric and does not take into account negative class predictions (Shmueli, 2019), whereas Cohen’s kappa is sensitive to class distributions (Widmann, 2020). Therefore, a more reliable performance measure was studied, i.e. MCC (also known as the phi coefficient or mean square contingency coefficient). MCC is a measure of association for two binary variables, i.e. it measures the differences between actual classes and predicted classes. The highest MCC was noted for both GNN and XGBoost technique with of value of 0.48. The lowest MCC score was noted for GRU-DNN with of value 0.41.

To understand the differences between these techniques with respect to erroneously classified compounds, we compared their false positive rate (FPR) and false negative rate (FNR). The FPR is the proportion of the negative class compounds that the classifier has assigned the positive class, whilst the FNR is the proportion of the positive class compounds for which classifier has assigned negative class. Based on the findings in Fig. 4, it was noted that XGBoost technique predicted lowest false positives (FP), and GNN technique showed lowest false negatives (FN). The GRU-DNN technique performed the worst among six, showing both the highest FPR and FNR.

A positive predictive value (PPV) describes the probability that, based on classifier’s positive prediction, a compound will truly belong to the positive class. Whereas a negative predicted value (NPV) describes the probability that, based on classifier’s negative prediction, a compound will truly belong to the negative class. The validation set’s highest PPV score of 0.29 was shown by both GNN and XGBoost. All techniques have validation set NPV scores equal to or above 0.99 (refer **SI** Table 2).

### Test set prediction outcome

All 20 constructed models per technique were used to assess test set compounds and results are described in Table 2 and **SI** Table 3. For the test set prediction, the 20 model ensemble from all six techniques have shown AUC ROC scores between 0.93 and 0.95. The XGBoost, GNN and SVM models have all shown AUC ROC scores equal to 0.95. The DNN and RF models have shown AUC ROC scores equal to 0.94 while GRU-DNN showed lowest test set AUC ROC score equals 0.93. Both GNN and XGboost models have showed highest BA scores which were equal to 0.90. The GRU-DNN model ranked last with a BA score of 0.87.

The XGBoost model showed highest MCC score of 0.48, GNN model ranked 2nd with a score of 0.47. The SVM model was ranked 3rd with a score of 0.45 whilst GRU-DNN model ranked last with a score equal to 0.41. Based on F1 score and Cohen’s kappa, the XGBoost model showed highest score, whereas the GNN model ranked 2nd. When we compared FPR and FNR of all the six models (see Fig. 4), it was evident that the XGBoost model predicted lowest false positives with FPR score equal to 0.07. The GNN model ranked 2nd with FPR score equals 0.08. In case of FNR, the GNN model performed best with lowest false negatives and with FNR score equals 0.12. The SVM and XGBoost models both have shown second lowest FNR score which was equal to 0.13. The GRU-DNN model showed worst performance in terms of FPR and FNR.

### Ranking of all techniques

The AUC ROC score of the validation was used as criteria to rank all six techniques in the beginning (see Fig. 5). The GNN was found to be the finest performing techniques with validation set AUC ROC scores equal to 0.96 and standard deviations in AUC ROC scores was equal to 0.0055 (see **SI** Table 2). The XGBoost model ranked second with AUC ROC score equals 0.95 with standard deviation of 0.0047. Further analysis showed that the XGBoost technique ranked first on the basis of F1 score, and Cohen’s kappa; while GNN techniques ranked second on these two metrics. Based on BA, the GNN was found to be the highest ranker. In case of incorrect predictions, the XGBoost ranked best for lowest FPR score, whereas GNN ranked 2nd. Meanwhile, based on FNR, the GNN technique ranked at the top with lowest FNR score, whereas the XGBoost ranked at the 3rd position from the top. Based on stability, we found XGBoost had lowest deviation in validation AUC ROC score of its 20 models with the standard deviation of 0.0047. In summary, we noted that based on kappa, f1 score and FPR scores the XGboost model was found to be the best performer whereas based on AUC ROC score, BA, sensitivity and FNR scores the GNN model was found to be the best performer.

From the drug safety aspect, it is important to filter out all risky hits early in the drug development pipeline. With this goal, we believe it is important for any *in silico* model to identify hERG active compounds than the inactive. The GNN model was found to have showed highest sensitivity along with lowest FN in comparison to all other five models.

When we compared model construction time for both top performing models, we found that in average training, the GNN required 3050 min of training time whereas XGBoost was built in 17 min. But when we did the same comparison for forward predictions, we found that GNN was more than 200 times faster than XGBoost. It was worth to invest our time in building GNN model, which for any future run, is extremely time saving. Fig. 6 shows this tradeoff as a linear extrapolation, where the forward prediction time of the GNN is about 0.003 s, while that of XGBoost is 0.626. The breakeven point is after screening about 300,000 compounds after the long training time of GNNs start becoming worth it. The forward prediction time of the XGBoost model is dominated by the calculation of descriptors and any descriptor-based model will suffer from this.

### Comparison with other published models

The predictive performance of the GNN model was compared with another existing published model. As presented in Table 3, the model described by Ogura *et al.* (Ogura et al., 2019) showed comparable performance to that of our model. This model showed a slightly higher test set AUC ROC score but a lower BA score than our model. Moreover, in comparison with the current model, the model from Ogura *et al.* showed higher number of false negatives that gave rise to a lower sensitivity of 0.67 for the test set prediction. The TP, FN, FP and TN values of the Ogura *et al.* model was 1987, 979, 410, and 83985, respectively. In this aspect, the current GNN model demonstrated better performance, showing a sensitivity equal to 0.88 and false negatives equal to 333. We would also like to point out that Ogura *et. al.* study considered whole training set to build their model, while our study required us to set aside few compounds for hyperparameter optimization and few for early stopping. Moreover, in case of the test set prediction, our GNN approach required processing of compound using a third-party toolkit (RDKit), which couldn’t process some 5638 test set compounds. Thus, our performance statistics was drawn from a smaller test set than that of Ogura *et. al*.’s study. Lastly, since there is lack of information whether Ogura *et al.*’s study tuned the class discretization threshold or not, therefore it is not sensible to compare our model with Ogura *et. al.*’s model based on BA and FN.

**Table 3:** Comparison of our model with another published model.

**Description**	**Our study**	**Ogura *et al.* (Ogura, Sato et al., 2019)**
Method	GNN	SVM
Descriptors	Graph embeddings	ECFP_4, 2-D, 3-D
Train set	203,853	203,841
Test set	81,728	87,361
BA_validation_	0.90	0.85
AUC-ROC score_validation_	0.96	–
BA_Test_	0.90	0.83
AUC-ROC score_Test_	0.95	0.96

From a practical point of view, we did not use any 3-D descriptors which require molecular geometry optimization that in turn would demand higher computational cost and time. Moreover, our approach did not involve any tedious descriptor selection procedures. Therefore, our approach was simpler, less time consuming, computationally cheap and relatively efficient, as compared to the work described by Ogura *et al.* (Ogura et al., 2019).

### External test set validation

To assess model’s performance on a new dataset, we obtained an external test set of 825 compounds from Karim *et al.* study (Karim et al., 2021). After removing 22 common compounds with our datasets, we were left with 803 compounds (refer **SI** Table 4). We validated our model using this external test set of 803 compounds and results are described in Table 4 and **SI** Table 5. The XGBoost model has shown highest AUC ROC score while SVM and DNN models have showed second highest AUC ROC score when validated using this external test set. The GNN and RF ranked third in this comparison. On the basis of BA, model XGBoost, SVM and DNN have shown equal performances with BA equal to 0.70. GNN, RF and GRU-DNN ranked second with BA equal to 0.67. In terms of sensitivity, the DNN model showed the highest sensitivity while on the basis of specificity, the GNN model showed the highest specificity. In case of FPR, the GNN model showed the lowest FPR rate while the DNN model showed the lowest FNR rate.

**Table 4:** Summary of the external test set prediction.

Technique	TN	FP	FN	TP	BA	AUC ROC score
GNN	452	282	18	51	0.67	0.75
XGBOOST	391	343	10	59	0.70	0.78
SVM	436	298	13	56	0.70	0.77
DNN	394	340	9	60	0.70	0.77
RF	396	338	14	55	0.67	0.75
GRU-DNN	409	325	16	53	0.67	0.72

TN = True Negative, FP = False Positive, FN = False Negative, TP = True Positive, BA = Balanced Accuracy.

In comparison to Karim *et al.*’s work, we found that our model performed equally well. The Karim *et al.*’s study divided the given test set into three different sets and stated BA for these three sets equal to 0.75, 0.75, and 0.81. For our work we have combined all these sets and removed overlapping compounds with respect to our training and test set, respectively. On our combined test set, our models show performances in the range of 0.67 to 0.70. It should be noted that, among those discarded overlapping 22 compounds, we found four compounds that had different assigned toxicity classes than those found in Karim *et al.*’s dataset (see **SI** Table 6). Therefore, problems regarding data quality, e.g. class assignments, cannot be ruled out as a cause for the observed differences in performance. Another factor that may have contributed to the differences in performance is the inability of our descriptor-free models in handling isomeric smiles.

### Feature importance analysis for the 1st random forest model

A RF consisted of hundreds of decision trees. Each decision tree is comprised of a set of internal nodes and leaves. During the binary decision tree building, a subsampled training set is, within each of its internal nodes, split into two groups based on a feature to separate similar classes of compounds. A Gini impurity is a parameter that determines how such node splitting should be optimally achieved. The mean decrease of impurity (MDI) is another important parameter that is used for identifying important features. MDI is the amount of the contribution of each feature toward the homogeneity of the internal nodes and leaves, aggregated across all trees of the RF. We have used the MDI parameter to identify the top 10 most significant descriptors within our 1st RF among 20 models (see Fig. 7). The two-sample Welch T-test was performed for all 10 descriptors, where all these descriptors were found statistically significant (refer Table 5).

**Table 5:** Welch T-test outcome for the top ten most important features from the 1st RF model.

**Descriptor**	**P-value**	**Mean descriptor value for negatives**	**Mean descriptor value for positives**	**Remark**
nBase	2.2e-16	0.0257	0.1000	Significant
ATS3i	2.2e-16	0.2693	0.3915	Significant
ATS5i	2.2e-16	0.2041	0.2986	Significant
ATS3pe	2.2e-16	0.2570	0.3652	Significant
ATS1i	2.2e-16	0.3408	0.4586	Significant
SsssN	2.2e-16	0.1836	0.3075	Significant
MID_C	2.2e-16	0.3868	0.5008	Significant
SdO	2.2e-16	0.2167	0.1389	Significant
nBondsD	2.2e-16	0.1850	0.1161	Significant
ATS3p	2.2e-16	0.2774	0.3881	Significant

### Scaffold-based analysis for the test set

To investigate, if some of our models have performed worse than others for any structural class, we have undertaken the Murcko’s scaffold-based analysis task. We have depicted four such scaffolds (i.e. Scaffold A, B, C and D) from the test set (see Fig. 8). The predictions associated with compounds containing these scaffolds have been listed in **SI** Table 7. Scaffold A and B were observed in the positive class test set compounds. Scaffold A containing compounds were erroneously predicted by several of our models, while scaffold B containing compounds were correctly predicted by all our models (refer **SI** Table 7).

Analyzing important descriptors of scaffold B containing test set compounds (i.e. correctly predicted positives), we found that their descriptor values were close to mean descriptor values for the entire positive class test set (refer Table 5, Table 6). In contrast, descriptor values of test set compounds containing scaffold A (i.e. erroneously predicted positives) were close to mean descriptor values for the entire negative class test set. This may be the reason why the latter were classified as negatives. Similar trends were observed in the case of scaffold C and D containing compounds where erroneously predicted compounds (scaffold C) showed descriptor values close to mean descriptor values of entire positive class test set (refer Table 5, Table 6).

**Table 6:** Top five important feature values for the test set compounds containing scaffolds A-D.

**Scaffold**	**Compoundindex**	**Actual class**	**Predicted class**	**Descriptors**				
				**nBase**	**ATS3i**	**ATS5i**	**ATS3pe**	**ATS1i**
A	254	1	0	0	0.23	0.17	0.24	0.33
	1282	1	0	0	0.22	0.17	0.22	0.33
	1714	1	0	0	0.27	0.24	0.27	0.4
B	371	1	1	0.1	0.35	0.25	0.33	0.42
	1352	1	1	0.1	0.35	0.25	0.32	0.42
	2551	1	1	0.2	0.45	0.35	0.41	0.53
	2565	1	1	0.1	0.46	0.36	0.42	0.53
C	3649	0	1	0.1	0.41	0.3	0.39	0.47
	17686	0	1	0.1	0.41	0.3	0.39	0.47
	44115	0	1	0.1	0.42	0.32	0.4	0.49
	53325	0	1	0.1	0.41	0.3	0.38	0.47
D	9920	0	0	0	0.18	0.16	0.2	0.26
	26991	0	0	0	0.2	0.18	0.21	0.29
	36012	0	0	0	0.2	0.19	0.22	0.29

### Factors affecting predictive performances of the currently described models

hERG Derived toxicity is not only limited to hERG-blockade but also depends upon pharmacokinetic factors (Lehmann et al., 2018). In this regard, our analysis was solely based on chemical description/representation and did not involve any pharmacokinetic parameters. There is a current debate concerning setting thresholds for hERG inhibition in terms of receptor interaction and which models are used to assay this, where thresholds used can range from 1 µM to 100 µM. The authors of the hERG dataset in the current study (Ogura et al., 2019) have used a 10 µM threshold for discretization. Moreover, several types of activities were converted into nanomolarity, therefore problems regarding data quality and compound categorization cannot be ruled out.

## Conclusions

Here, we have successfully benchmarked six ML techniques for the prediction of hERG-derived toxicity. In conclusion, traditional ML methods such as RF, SVM, DNN, and XGBoost performed equally well as compared to advanced deep learning techniques such as GNN and GRU-DNN. The ranking based on predictive performances merited the GNN as a winning technique, which is an advanced deep learning technique. The main advantages associated with the GNN technique is that it does not need any precalculated descriptors to begin with, it does not require any feature engineering such as descriptor filtering/selection, and lastly it has the least human interventions and allows for comprehensive automation. We believe our top performing models may serve as a promising tool for both academic institutions as well as pharmaceutical industries in screening compounds for hERG-derived toxicity. The advanced deep learning models provided here are trained with the large set of toxicologically relevant compounds and could best serve as pretrained models for AI community for toxicology related research. The approaches detailed in the current study may also serve as a basis for further automating computational predictions of key toxicity endpoints with the application of AI.

## Funding

This work has been funded by Research Institutes of Sweden RISE’s internal project “AI-TOX” (grant no. KFT SK-2021). Partial funding has been received from the “Safe and Efficient Chemistry by Design (SafeChem)” project funded by the 10.13039/501100001729Swedish Foundation for Strategic Environmental Research, MISTRA (grant no. DIA 2018/11).

## CRediT authorship contribution statement

**Erik Ylipää:** Conceptualization, Methodology, Formal analysis, Software, Validation, Visualization, Investigation, Writing – original draft. **Swapnil Chavan:** Conceptualization, Data curation, Methodology, Formal analysis, Software, Validation, Visualization, Investigation, Writing – original draft. **Maria Bånkestad:** Conceptualization, Methodology, Formal analysis, Software, Validation, Visualization, Investigation, Writing – original draft. **Johan Broberg:** Methodology, Software, Investigation, Writing – review & editing. **Björn Glinghammar:** Supervision, Writing – review & editing. **Ulf Norinder:** Supervision, Writing – review & editing. **Ian Cotgreave:** Supervision, Funding acquisition, Writing – review & editing.

## Declaration of Competing Interest

The authors declare the following financial interests/personal relationships which may be considered as potential competing interests: [Prof. Dr. Ian Cotgreave reports financial support was provided by Swedish Foundation for Strategic Environmental Research.].

## Data Availability

Data is allready available and link has been provided in the reference list

## Appendix A Supplementary material

Supplementary Data 1 Tuned AUC ROC score thresholds for each model for each technique have been listed in . Detailed prediction statistics for validation, test and external-test set have been provided in , respectively. The SMILES and activity classes of the external test set have been provided in . The Ogura et al.’s training and test set compounds where conflicting toxicity classes was found with respect to Karim et al.'s dataset, are listed in . The predictions for compounds containing scaffolds A-D are listed in .
